# Improved Protective Efficacy of a Species-Specific DNA Vaccine Encoding Mycolyl-Transferase Ag85A from *Mycobacterium ulcerans* by Homologous Protein Boosting

**DOI:** 10.1371/journal.pntd.0000199

**Published:** 2008-03-19

**Authors:** Audrey Tanghe, Jean-Pierre Dangy, Gerd Pluschke, Kris Huygen

**Affiliations:** 1 Mycobacterial Immunology, IPH-Pasteur Institute Brussels, Brussels, Belgium; 2 Department of Molecular Immunology, Swiss Tropical Institute, Basel, Switzerland; University of Tennessee, United States of America

## Abstract

Vaccination with plasmid DNA encoding Ag85A from *M. bovis* BCG can partially protect C57BL/6 mice against a subsequent footpad challenge with *M. ulcerans*. Unfortunately, this cross-reactive protection is insufficient to completely control the infection. Although genes encoding Ag85A from *M. bovis* BCG (identical to genes from *M. tuberculosis*) and from *M. ulcerans* are highly conserved, minor sequence differences exist, and use of the specific gene of *M. ulcerans* could possibly result in a more potent vaccine. Here we report on a comparison of immunogenicity and protective efficacy in C57BL/6 mice of Ag85A from *M. tuberculosis* and *M. ulcerans*, administered as a plasmid DNA vaccine, as a recombinant protein vaccine in adjuvant or as a combined DNA prime-protein boost vaccine. All three vaccination formulations induced cross-reactive humoral and cell-mediated immune responses, although species-specific Th1 type T cell epitopes could be identified in both the NH_2_-terminal region and the COOH-terminal region of the antigens. This partial species-specificity was reflected in a higher—albeit not sustained—protective efficacy of the *M. ulcerans* than of the *M. tuberculosis* vaccine, particularly when administered using the DNA prime-protein boost protocol.

## Author Summary

Buruli ulcer (BU) is an infectious disease characterized by deep, ulcerating skin lesions, particularly on arms and legs, that are provoked by a toxin. BU is caused by a microbe belonging to the same family that also causes tuberculosis and leprosy. The disease is emerging as a serious health problem, especially in West Africa. Vaccines are considered to be the most cost-effective strategy to control and eventually eradicate an infectious disease. For the moment, however, there is no good vaccine against BU, and it is still not fully understood which immune defence mechanisms are needed to control the infection. The identification of microbial components that are involved in the immune control is an essential step in the development of an effective vaccine. In this paper, we describe the identification of one of these microbial components, i.e., antigen 85A, a protein involved in the integrity of the cell wall of the microbe. Our findings obtained in a mouse model now need to be extended to other experimental animals and later to humans. Combination with a vaccine targeting the toxin may be a way to strengthen the effectiveness of the vaccine.

## Introduction

Buruli ulcer (BU), also known as Bairnsdale ulcer, is an infectious, necrotizing skin disease caused by *Mycobacterium ulcerans* (*M. ulcerans*) occurring mostly in tropical and subtropical areas. Cases have been reported in several countries in West and Central Africa, in Central and South America, in Southeast Asia and in Australia. BU is emerging as a serious health problem, especially in West Africa, where it is the third leading cause of mycobacterial disease in immunocompetent people, after tuberculosis and leprosy. In some countries in Africa, thousands of cases occur annually and in these areas BU has supplanted leprosy to become the second most important human mycobacterial disease. The natural history of *M. ulcerans* infection and subsequent development of BU is not completely elucidated. *M. ulcerans* bacteria have been found in endemic areas in stagnant water or slowly moving water sources and in aquatic snails and carnivorous insects [1],[2]. So far, person to person transmission has not been reported. The infection causes initially a painless nodular swelling which can eventually develop into an extensive necrotizing lesion. *M. ulcerans* has the particularity to produce a family of toxin molecules, the so-called mycolactone (ML), polyketides that can suppress the immune system and destroy skin, underlying tissue and bone, causing severe deformities [3]–[5]. ML suppresses the *in vitro* TNF-α production by murine macrophages infected with *M. ulcerans* (4) and it strongly affects the maturation and the migratory properties of DC [5]. On the other hand, ML does not seem to affect the production of the inflammatory cytokine MIP-2, involved in the recruitment of neutrophils (4). *M. ulcerans* has an initial intracellular infection stage but virulent ML producing strains induce apoptosis of the infected cells and can subsequently be found extracellularly [3],[6]. Only few *Mycobacterium* species produce mycolactone toxins [7]. *M. ulcerans* isolates from different geographical areas produce different types of mycolactone, i.e. mycolactone A/B, C, D, E and F [8],[9].

The nature of immune protection against *M. ulcerans* infection remains unclear. In general, resistance to intracellular bacteria is primarily mediated by T cells with pivotal roles of Th1 type cytokines IFN-γ and TNF-α and this apparently is the case for *M. ulcerans* infection as well [10]. Progression of active Buruli ulcer is characterized by gradual down regulation of systemic and local Th1 type immune responses. Peripheral blood mononuclear cells from Buruli ulcer patients show reduced lymphoproliferation and IFN-γ production in response to specific stimulation with *M. ulcerans* [11]–[13]. Reduced IFN-γ response does not seem to be caused by decreased interleukin-12 production [14]. Also, semi-quantitative RT-PCR analysis demonstrated high IFN-γ and low IL-10 levels in early, nodular lesions whereas low IFN-γ and high IL-10 mRNA levels are observed in late ulcerative lesions [13]. Using a similar RT-PCR comparison of granulomatous versus non-granulomatous lesions, Phillips *et al* demonstrated higher expression of IL-12p35, IL-12p40, Il-15, IL-1β and TNF-α in patients from the former group and higher expression levels of IL-8 (human homologue of MIP-2) in the latter group [15]. Finally, Kiszewski *et al* have also confirmed that in ulcerative lesions without granuloma, there is increased expression of IL-10 and higher bacillary counts. [16].

It is not yet clear whether antibodies play a protective role against BU but the humoral immune response during *M. ulcerans* infection may be useful for serodiagnosis of BU. In contrast to tuberculosis and leprosy, immunoglobulin IgG antibody production against *M ulcerans* can be found even in early stages of infection [17]. IgG antibodies cannot be used to readily discern between patients and family controls, but primary IgM antibody responses against *M. ulcerans* culture filtrate proteins can be detected in sera from 85% of confirmed BD patients and only in a small proportion in sera from healthy family controls [18]. Antibody responses against the *M. ulcerans* homologue of the *M. leprae* 18-kDa small heat shock protein -that has no homologues in *M. bovis* and *M. tuberculosis*- can be used as serological marker for exposure to *M. ulcerans* [19].

BU results in considerable morbidity. Because of the late detection of the disease, treatment is principally by excision of the lesion, sometimes necessitating skin grafting [20]. WHO is currently recommending combined rifampicin and streptomycin treatment of nodules for eight weeks in the hope of reducing the need for surgery [21],[22]. Unfortunately, there is no specific vaccine against BU for the moment [23]. *M. bovis* BCG (Bacille Calmette et Guérin) vaccine, used for the prevention of tuberculosis, has been reported to offer a short-lived protection against the development of skin ulcers [24]–[26] and to confer significant protection against disseminated cases of BU, e.g. osteomyelitis, both in children and in adults [27],[28]. The precise *M. ulcerans* antigens that induce a protective immune response are poorly defined. The complete genome sequence of *M. ulcerans* has recently been published and will hopefully help to advance research and identification of relevant genes [29]. The 65 kD heat shock protein is expressed in considerable amounts by *M. ulcerans* bacilli *in vitro* and *in vivo*, and is immunogenic for both B and T cells in mice. Nevertheless, vaccination of mice with plasmid DNA encoding Hsp65 from *M. leprae*, having 96% sequence identity with Hsp65 from *M. ulcerans*, limited only weakly the progression of experimental *M. ulcerans* infection in tail [30]. We have previously reported that vaccination with BCG or with plasmid DNA encoding Ag85A from *M. bovis* BCG can partially protect B6 mice against footpad challenge with *M. ulcerans* [31]. Antigen 85 is a major secreted component in the culture filtrate of many mycobacteria such as *M. bovis* BCG, *M. tuberculosis* and *M. avium* subsp. *paratuberculosis* [32]. The antigen 85 complex (Ag85) of *M. tuberculosis* is a family of three proteins, Ag85A, Ag85B and Ag85C, which are encoded by three distinct but highly paralogous genes and that display an enzymatic mycolyl-transferase activity, involved in cell wall synthesis [33],[34]. Members of the Ag85 family rank among the most promising tuberculosis vaccine candidates, and are actually being tested in clinical trials, formulated as Hybrid-1 fusion protein of Ag85B with ESAT-6 or as recombinant Modified Vaccina Ankara virus encoding Ag85A booster vaccine of BCG [35],[36]. We have previously sequenced the gene encoding Ag85A from *M. ulcerans* and reported that it shares 84.1% amino acid sequence identity and 91% conserved residues with the gene encoding Ag85A from *M. tuberculosis* (which is identical to the Ag85A gene of *M. bovis* BCG) [31]. The genes encoding Ag85B and Ag85C of *M. ulcerans* have recently been sequenced as well and – as for *M. tuberculosis*- were localized on different loci in the genome [29].

Here, we report on a comparison of the immunogenicity and protective efficacy of vaccines encoding Ag85A from *M. tuberculosis* and from *M. ulcerans.* Vaccines were administered as plasmid DNA, purified protein in adjuvant or in a DNA prime-protein boost protocol. We and others have previously reported that DNA priming followed by protein boosting is an effective means to increase the potential of DNA vaccines [37]–[40].

## Materials and Methods

### Mice

C57BL/6 mice were bred in the Animal Facilities of the IPH-Pasteur Institute Brussels, from breeding couples originally obtained from Bantin & Kingman (UK). Mice were 8–10 weeks old at the start of the experiments. Female mice were used for immune analysis and male mice for the protection studies. This study has been reviewed and approved by the local Animal Ethics Committee (file number 030212/05).

### Mycobacterial strains

Virulent *M. ulcerans* type 1 strain 04-855 from a Benin patient was isolated at the Institute for Tropical Medicine in Antwerp, Belgium. Bacteria grown on Löwenstein-Jensen medium were maintained and amplified *in vivo* in footpad of the mice. *M. bovis* BCG strain GL2 was grown for 2 weeks as a surface pellicle at 37°C on synthetic Sauton medium and homogenized by ball mill as described before [41].

### Plasmid DNA constructions

Plasmid DNA encoding the mature 32 kD Ag85A from *M. tuberculosis* in V1J.ns-tPA vector was prepared as described before [31],[42]. The gene encoding Ag85A from *M. ulcerans* was amplified by PCR without its mycobacterial signal sequence using *Bgl*II restriction site containing primers and ligated into the same V1J.ns-tPA vector. The primers used were 5′-GGAAGATCTTGAGCGCTTGGTACTAGGC-3′ (forward) and 5′-GGAAGATCTTTTCGCGGCCGGGCCTGCCGGTGGA-3′ (reverse). In these plasmids the Ag 85A gene is expressed under the control of the promoter of IE1 antigen from cytomegalovirus, including intron A and it is preceded by the signal sequence of human tissue plasminogen activator.

### Recombinant Ag85A proteins

Hexa-histidine tagged Ag85A protein from *M. tuberculosis* was purified from recombinant *E. coli* as described before [43]. The gene encoding the mature Ag85A protein from *M. ulcerans* was amplified by PCR from V1J.ns.tPA-85A vector. The primers used were 5′-CGCGGATCCGCGTTTTCGCGGCCGGGCCTGCCGTGGAA-3′ (forward) and 5′-CCCAAGCTTGGGCTAGGCGCCCTGGGTGTCACCG-3′ (reverse) with respectively *Bam*HI and *Hind* III restriction sites. Ag85A gene was amplified without its mycobacterial signal sequence. Cloning in expression vector pQE-80L (QIAGEN), containing an NH_2_-terminal histidine-tag coding sequence, and purification were performed as described before [32]. Briefly, positives clones were screened on LB-ampicillin medium after ligation of the gene in the vector and transformation of *E. coli* DH5α cells. For expression, Top-10F' *E. coli* (Invitrogen) cells were transformed with plasmid encoding the 85A sequence. Recombinant protein was purified by immobilized metal affinity chromatography (IMAC) using gravity flow. The endotoxin level measured with the LAL kinetic chromogenic assay, was inferior to 10 EU/ml (endotoxin units per millilitre) or 0.03 EU/µg of purified protein (Cambrex Bioscience, New Jersey, America).

### Peptide synthesis

Peptides spanning the entire mature 295 amino-acid Ag85A sequence of *M. tuberculosis* were synthesized as 20-mers, with the exception of the 18-mer spanning aa 35–53 and the 21 mer-peptide spanning amino acids 275–295 [44]. Peptides spanning the entire 294-amino acid Ag85A sequence of *M. ulcerans* were synthesized as 20-mers. All peptides were purchased from Ansynth Service B.V., The Netherlands.

### Vaccination protocols

In experiment 1, B6 mice were anesthesized by intraperitoneal injection of ketamine-xylazine and injected three times intramuscularly (i.m) in both quadriceps muscles with 2×50 µg of control V1J.ns-tPA (empty vector), V1J.ns-tPA-Ag85A DNA from *M. ulcerans* or from *M. tuberculosis* (abbreviated as Ag85A-DNA Mu and Ag85A-DNA Mtb in the figures). For protein immunization, mice were injected three times subcutaneously (s.c) in the back with 10 µg of purified recombinant Ag85A (abbreviated as rec85A-Mu and rec85A-Mtb in the figures), emulsified in Gerbu adjuvant, i.e. water miscible, lipid cationic biodegradable nanoparticles, completed with immunomodulators and GMDP glycopeptide (GERBU Biochemicals). For the DNA prime-protein boost, mice were immunized twice i.m. with Ag85A DNA from *M. ulcerans* or from *M. tuberculosis* and boosted s.c. with 20 µg of recombinant Ag85A protein respectively from *M. ulcerans* or *M. tuberculosis* in Gerbu adjuvant (abbreviated as Ag85A-DNA/recMu and Ag85A-DNA/recMtb in the figures). All mice received the two first injections at 3 week intervals and the third injection was given two months later. For BCG vaccination, mice were injected intravenously, in a lateral tail vein, at the time of the first DNA injection with 0.2 mg (corresponding to 10^6^ CFU) of freshly prepared live *M. bovis* BCG [41].

In experiment 2, B6 mice were injected intramuscularly (i.m) three times, at 3 weeks intervals, in both quadriceps with 2×50 µg of control V1Jns.tPA DNA or plasmid DNA encoding 85A from *M. ulcerans* or from *M. tuberculosis*. For protein immunization, mice were injected three times subcutaneously (s.c) in the back with 10 µg of purified recombinant Ag85A from *M. ulcerans* or from *M. tuberculosis*, emulsified in monophosphoryl lipid A (MPL-A) from *Salmonella enterica* serovar Minnesota (Ribi ImmunoChem Research, Hamilton, Mont)) solubilized in triethanolamine. For the DNA prime-protein boost, mice were immunized twice i.m. with Ag85A DNA from *M. ulcerans* or from *M. tuberculosis* and boosted s.c. with 20 µg of purified recombinant Ag85A protein respectively from *M. ulcerans* or from *M. tuberculosis* in MPL-A.

### Infection

Naïve and vaccinated B6 mice were infected with *M. ulcerans* 3 months (Exp1) or 6 weeks (Exp2) after the last vaccination. 10^5^ acid fast bacilli (AFB), obtained by *in vivo* passage in footpad, were injected in the right footpad of the vaccinated mice. The number of bacilli injected, suspended in Dubos Broth Base medium (Difco), was determined by counting under a microscope after Ziehl Neelsen staining. Viability of the *M. ulcerans* inoculum was checked by plating on 7H11 Middlebrook agar, supplemented with oleic-acid-albumin-dextrose-catalase enrichment medium. Yellow colonies were counted after 8 weeks of incubation at 32°C. The number of Colony Forming Units corresponded to the number of AFB.

### Cytokine production

Vaccinated mice were sacrificed 3 weeks after the third immunization (Experiment 1). Spleens were removed aseptically and homogenized in a loosely fitting Dounce homogenizer. Leucocytes (4×10^6^ WBC/ml) from four mice per group were cultivated at 37°C in a humidified CO_2_ incubator in round-bottom micro well plates individually or pooled (as indicated) and analyzed for Th1 type cytokine response to purified recombinant his-tagged Ag85A (5 µg/ml), and synthetic peptides from *M. ulcerans* or *M. tuberculosis* (10 µg/ml). Supernatants from at least three wells were pooled and stored frozen at −20°C. Cytokines were harvested after 24 h (IL-2) and 72 h (IFN-γ), when peak values of the respective cytokines can be measured.

### IL-2 assay

Interleukin-2 (IL-2) activity was determined in duplicate on 24 h culture supernatants using a bio-assay with IL-2 dependent CTLL-2 cells as described before [45]. IL-2 levels are expressed as mean counts per minute (cpm). Assay sensitivity is 10 pg/ml. A typical international standard curve of this assay has been published before [46].

### IFN-γ assay

Interferon-γ (IFN-γ) activity was quantified by sandwich ELISA using coating antibody R4-6A2 and biotinylated detection antibody XMG1.2 obtained from Pharmingen. The standard murine recombinant IFN-γ used was obtained from R&D. The sensitivity of the assay is 10 pg/ml.

### ELISA

Sera from immunized mice were collected by tail bleeding 3 weeks after the third vaccination. Levels of *M. ulcerans* specific total anti-Ag85A Igκ antibodies (Abs) were determined by direct enzyme-linked immunosorbant assay (ELISA) in sera from individual mice (four/group). The concentration of Ab was expressed by the optical density at a dilution of 1/100 of the sera. For isotype analysis, peroxidase-labeled rat anti-mouse immunoglobulin G1 (IgG1) and IgG2b (Experimental Immunology Unit, Université Catholique de Louvain, Brussels, Belgium) were used. Isotype titers were expressed as dilution endpoints (last serum dilution with an optical density (OD) value higher than a cut-off OD value calculated from the OD value plus three standard deviations (SD) of the secondary antibody only [42].

### Protection analysis

In experiment 1 (Gerbu adjuvant), protection was evaluated by enumeration of Acid Fast Bacilli (AFB) nine weeks after footpad infection. Briefly, the skin and bones were removed from infected foot pad. Tissues were homogenized in a Dounce homogenizer and suspended in 2 ml of Dubos broth based medium containing glass bead. The number of AFB in 20 fields (surface of 1 field: 0.037994 mm^2^×20 with the 22 mm ocular diameter used) was counted on microscope slides after Ziehl-Neelsen staining. In experiment 2 (MPL-A adjuvant), protection was evaluated by monitoring foot pad swelling after *M. ulcerans* infection. The swelling was measured with a calibrated Oditest apparatus with a resolution of 0.01 mm as described previously [47]. Animals were euthanized when footpad swelling exceeded 4mm according to the rules of the local ethical commission.

### Statistical analysis

For cytokine production analysis, antibody production and AFB counting, statistical analysis was made according to one-way ANOVA test. Subsequent multiple comparison between the 7 different groups of animals and the antigens used was made by a Tukey's correction test. Statistical results are represented in the figure by *** (P<0.001), ** (P<0.01) and * (P<0.05). For the comparison of survival curves, logrank test was used.

## Results

### Partially species-specific Th1 type cytokine production in spleen cell cultures from B6 mice vaccinated with Ag85A-DNA, Ag85A protein or with a DNA prime-protein boost

**Figure 1:**
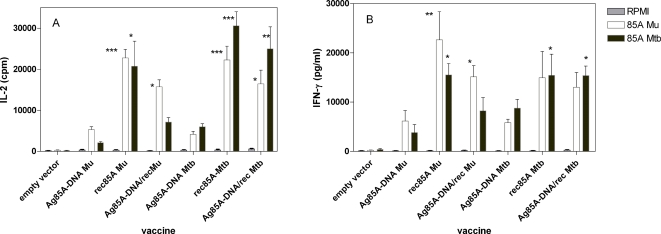
Spleen cell IL-2 (A) and IFN-γ (B) responses to RPMI medium (grey bars), recombinant Ag85A from *M. ulcerans* (white bars) and *M. tuberculosis* (black bars) in B6 mice vaccinated with Ag85A DNA, Ag85A protein or Ag85A DNA boosted with protein in Gerbu adjuvant, 3 months after the third immunization. Cytokines levels tested on 24 h (IL-2) and 72 h (IFN-γ) culture supernatant of 4 mice tested individually/group. Data presented as means±SDs intra-assay. Statistically significant results as compared to the DNA vaccinated *M. ulcerans* or *M. tuberculosis* groups are represented in the figure by *** (P<0.001), ** (P<0.01) and * (P<0.05). The first group of comparison take all of the vaccinated mice compared to the DNA vaccinated mice in response to recombinant Ag85A from *M. ulcerans*. The second group of comparison take all of the vaccinated mice compared to the DNA vaccinated mice in response to recombinant Ag85A from *M. tuberculosis.*

Spleen cells from mice vaccinated with the three different vaccine formulations produced significant levels of IL-2 (Figure 1A) and IFN-γ (Figure 1B) after *in vitro* stimulation with purified recombinant Ag85A from *M. ulcerans* or from *M. tuberculosis.* As expected from the 91% sequence similarity between both antigens, highly cross-reactive immune responses were observed, mice vaccinated with *M. ulcerans* vaccines recognizing the *M. tuberculosis* antigen and vice versa. Nevertheless, a certain level of species specificity was observed, particularly in the IL-2 responses. Confirming previous results obtained with a *M. tuberculosis* DNA vaccine [37], boosting plasmid DNA vaccinated mice with purified *M. ulcerans* protein increased significantly Ag 85A specific IL-2 and IFN-γ responses.

### *M. ulcerans* Ag85A specific antibody production in mice vaccinated with Ag85A-DNA, Ag85A protein or with a DNA prime-protein boost

**Figure 2:**
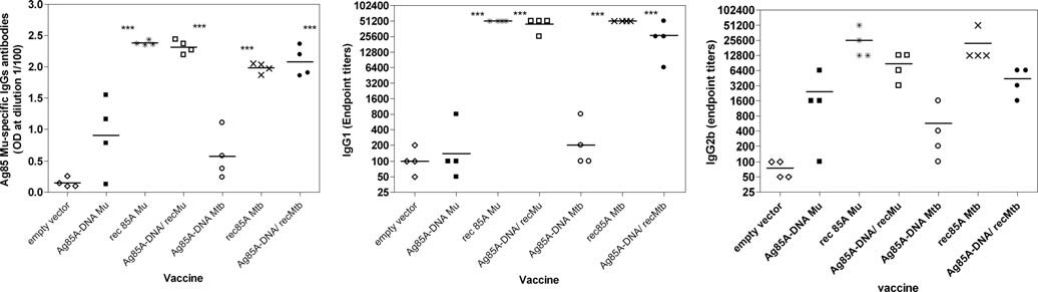
Ag85 *M. ulcerans* specific total IgG, IgG1 and IgG2b antibody levels in B6 mice. Protein emulsified in Gerbu adjuvant. Data represent individual antibody levels (4 mice/group), mean value is indicated by the horizontal bars. * statistical analysis showing each group with a significant difference. For IgG1, groups c, d, f and g have a significative difference with the others groups. For IgG2b, only rec 85A Mu was significatively different from group a and e.

Significant cross-reactive antibody responses were induced against Ag85A from *M. ulcerans* (and from *M. tuberculosis*, data not shown) in mice vaccinated with the *M. ulcerans* and *M. tuberculosis* vaccines (Figure 2).

Antibody responses in DNA vaccinated mice demonstrated considerable individual variation, and were markedly increased by the protein boost. Vaccination with purified protein in Gerbu adjuvant was also very effective in inducing high level antibody production. DNA vaccination induced very little IgG1 isotype antibodies but biased predominantly an IgG2b isotype response, confirming the well known Th1 inducing properties of intramuscular plasmid DNA. In contrast, vaccination with protein emulsified in Gerbu adjuvant induced antibodies of both IgG1 and of IgG2b isotype. Confirming previous findings, DNA prime- protein boost vaccination resulted in increased and less variable antibody titers of both isotypes [37]. Vaccination with recombinant 85A protein or with the DNA prime /protein boost protocol induced significantly higher levels of total IgG and IgG1 antibodies as compared to plasmid DNA vaccination alone (P<0.001).

### Identification of species-specific H-2^b^ restricted Th1 T cell epitopes in *M. ulcerans* Ag85A

**Figure 3:**
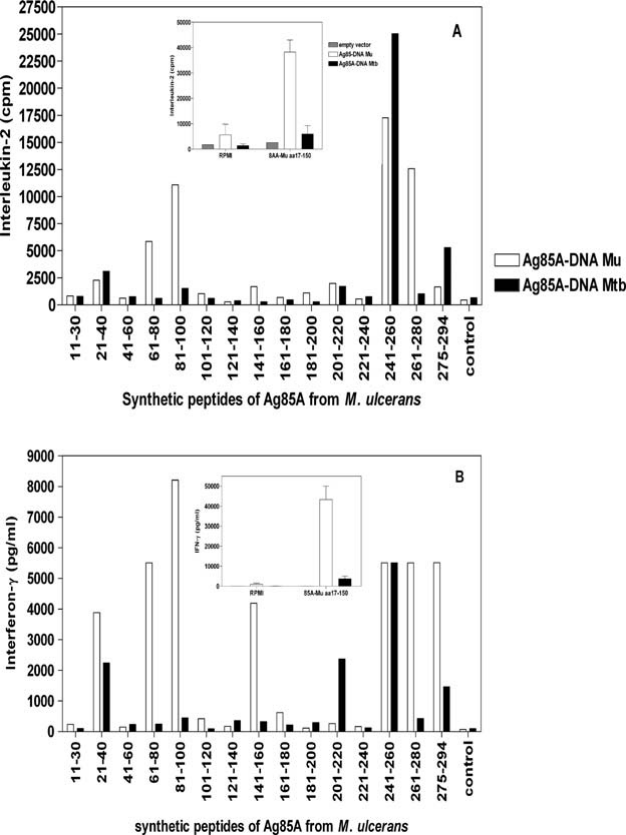
Spleen cell IL-2 (A) and IFN-γ (B) responses to whole Ag85A- *M. ulcerans* and its synthetic peptides, as tested on a pool of six B6 mice vaccinated 3 times at three weeks interval with Ag85A-DNA *M. ulcerans* (white bars) or Ag85A-DNA *M. tuberculosis* (black bars). Graph in insert represents the cytokine production in response to partial Ag85A *M. ulcerans* aa 17–150. Unstimulated cells (grey bars). Data of insert represent mean±SD values of 3 mice tested individually.

Despite the high level of sequence similarity (91%) between Ag85A from *M. tuberculosis* and *M. ulcerans* but in view of the partial species-specific Th1 type immune responses observed in the previous experiment, we decided to characterize the H-2^b^ restricted immunodominant T cell epitopes, using synthetic 20-mer peptides spanning the entire mature sequence of Ag85A from *M. ulcerans* and from *M. tuberculosis.* Figure 3 shows the IL-2 and IFN-γ production induced in response to *M. ulcerans* peptides in mice vaccinated with *M. ulcerans* DNA (white bars) or *M. tuberculosis* DNA (black bars). Spleen cells from B6 mice vaccinated with *M.ulcerans-*Ag85A DNA produced significant levels of IL-2 (Figure 3A) and IFN-γ (Figure 3B) when stimulated with *M. ulcerans* peptides both from the NH_2_-terminal and COOH-terminal part of the protein, whereas IL-2 and IFN-γ responses of B6 mice vaccinated with the *M. tuberculosis* plasmid were almost exclusively directed against *M. ulcerans* peptide spanning aa 241–260. *M. ulcerans* DNA vaccinated mice also recognized this peptide very effectively. Responses against the NH_2_-terminal peptides spanning aa 61–80 and 81–100 of *M. ulcerans-*Ag85A were only observed in *M. ulcerans* DNA vaccinated mice, indicating that this NH_2_-terminal region was responsible for the partial species-specificity. This confirmed a previous finding (Inserts in Figures 3A and 3B) on species-specific T cell responses induced following *in vitro* stimulation with a purified, partial *M. ulcerans* Ag85A protein, spanning aa 17–150 in mice vaccinated with DNA encoding Ag85A from *M. ulcerans* or *M. tuberculosis*. IL-2 and IFN-γ responses against *M. ulcerans* peptide spanning aa 261–280 were also species-specific and only detected in mice immunized with the *M. ulcerans* vaccine.

**Figure 4:**
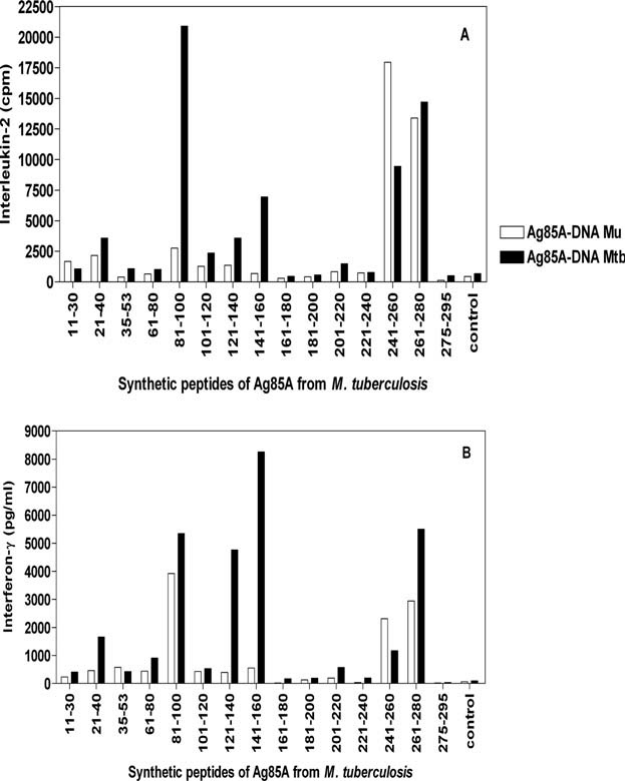
Spleen cell IL-2 (A) and IFN-γ (B) responses to whole Ag85A-*M. tuberculosis* and its synthetic peptides in a pool of six B6 mice vaccinated, at three weeks interval with Ag85A-DNA *M. ulcerans* (white bars) or Ag85A-DNA *M. tuberculosis* (black bars).

Responses against *M. tuberculosis* peptides showed a reciprocal pattern (Figure 4). Confirming previous findings [37] *M. tuberculosis* peptide spanning aa 261–280 was very well recognized in *M. tuberculosis* DNA vaccinated mice (Figure 4A and 4B). It was also recognized by *M. ulcerans* vaccinated mice. Both DNA vaccinated groups also reacted against *M. tuberculosis* peptide spanning aa 241–260, previously found to contain the immunodominant H-2^b^ restricted epitope recognized in BCG vaccinated and *M. tuberculosis* infected B6 mice (10). Responses against this *M. tuberculosis* peptide were even higher in *M. ulcerans* than in *M. tuberculosis* DNA vaccinated mice. IFN-γ responses against *M. tuberculosis* peptides spanning aa 121–140 and 141–160 were only observed in mice vaccinated with the *M. tuberculosis* DNA, whereas a cross-reactive immune responses was found against *M. tuberculosis* peptide spanning aa 81–100. A sequence comparison of identified immunodominant Th1 peptides of Ag85A from *M. ulcerans* and from *M. tuberculosis*, showing conserved and non-conserved amino acid changes is presented in Table 1.

**Table 1:** Immunodominant peptides of Ag 85 A from *M. ulcerans* and *M. tuberculosis*.

Peptides : aa	Sequences of *M. ulcerans* *	Sequences of *M. tuberculosis*
21–40	**N**IKVQFQSGGANSPALYLLD	**D**IKVQFQSGGANSPALYLLD
61–80	yyqsg**i**svampvggqssfys	YDQSG**L**SVVMPVGGQSSFYS
81–100	dwynpacgkagcttykwetf	DWYQPACGKAGCQTYKWETF
141–160	dqfvy**s**g**sl**salldpsqg **i**g	QQFVY**A**G**AM**SGLLDPSQA **M**G
240–259	FQAAYNAAGGHNAV**WN**FDD**N**	85A :FQDAYNAGGGHNGV**FD**FPD**S**
		85B :FQDAYNAAGGHNAVFNFPPN
261–280	thsweywgaqlnam**r**pdlqh	THSWEYWGAQLNAM**K**PDLQR
275–294	**r**pdlqhtlgatpntgdtqga	**K**PDLQRALGATPNTGPAPQGA

*: Bold Amino Acids are high conserved amino acid differences (determined according to the Needleman-Wunsh criterion) and Amino Acids underlined are the non-conserved aa difference in comparison to the Ag85A peptides of *M. tuberculosis*.

### Reduced *M. ulcerans* replication in footpad of mice vaccinated with Ag85A-DNA, Ag85A protein and with a DNA prime-protein boost

**Figure 5:**
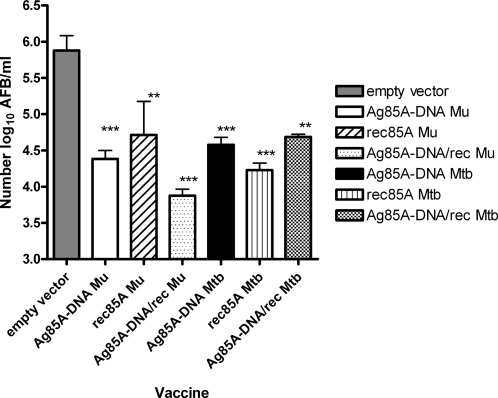
Mycobacterial multiplication in mice infected with *M. ulcerans* strain 04-855. Mice infected three months after the last vaccination (protocol 1 using Gerbu adjuvant). Results are mean±SD of AFB/ml of 4 individual mice, expressed in Log_10_. * p value as compared to mean log_10_ value obtained in control DNA mice infected with *M. ulcerans.*

Mice were challenged three months after the third vaccination with 10^5^ AFB of *M. ulcerans* in the footpad. Nine weeks later, when a significant swelling of the footpad appeared in the control mice vaccinated with empty vector, all animals were sacrificed and the number of AFB in the infected footpad was determined by Ziehl-Neelsen staining. As shown in Figure 5, a significant and strong reduction in the number of *M. ulcerans* AFB was observed in mice previously immunized with all three types of vaccine. Vaccination with specific *M. ulcerans* antigen using the DNA prime-protein boost protocol with Gerbu adjuvant conferred the highest protection with an almost one-hundred fold reduction in number of AFB as compared to the control group. This protection was comparable in magnitude to the protection conferred by the BCG vaccine. Difference between the vaccinated groups was not significant (ANOVA test; p>0.05).

### Prolonged survival after *M. ulcerans* challenge in mice vaccinated with Ag85A-DNA or with a DNA prime-protein boost

**Figure 6:**
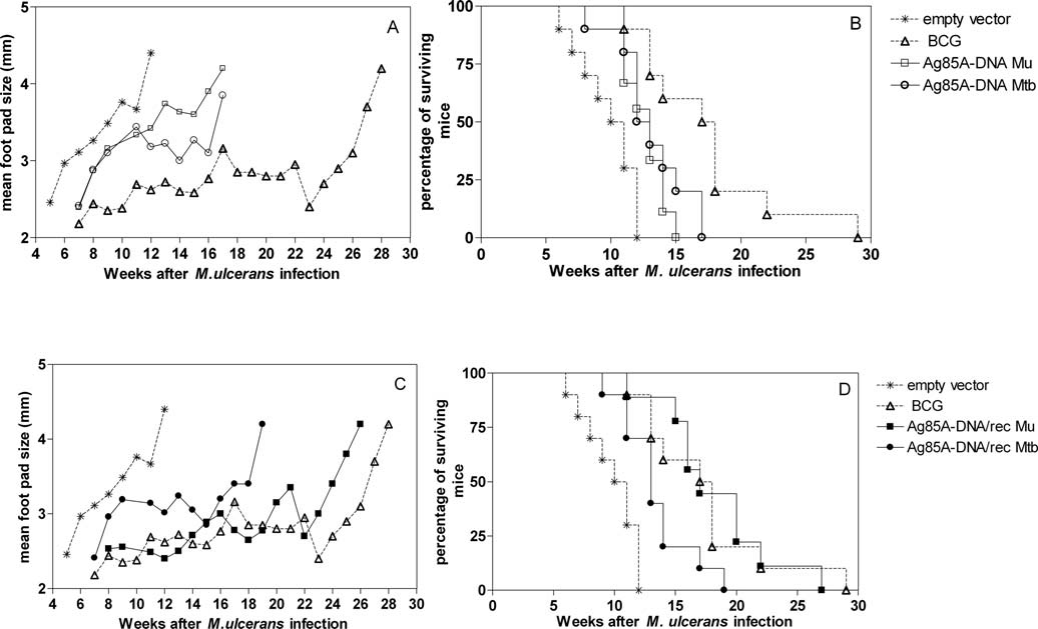
Evaluation of footpad size (A and C) and Survival curves (B and D) of vaccinated B6 mice after *M. ulcerans* infection with 10^5^ AFB in the right footpad. Experiment 2 using MPL-A as adjuvant and 6 weeks as rest between vaccination and infection. Mice were vaccinated with empty vector (* *n* = 9), with 0.2 mg of BCG (▵ *n* = 10), with Ag85-DNA from *M. ulcerans* (□ *n* = 9), with DNA encoding 85A from *M. tuberculosis* (○ *n* = 10), with Ag85A DNA-*M. ulcerans* boosted with the Ag85A protein from *M. ulcerans* (▪ *n* = 9), or with DNA encoding Ag85A boosted with Ag85 protein from *M. tuberculosis* (• *n* = 10).

In a second experiment, protective efficacy of the vaccines was determined by weekly monitoring appearance and size of footpad swelling and survival as previously reported [47]. Mice were euthanized when footpad swelling was >4 mm. In this experiment mice were challenged with 10^5^ AFB of *M. ulcerans* 04-855 at 6 weeks after the last immunization. The evolution of footpad swelling is shown in Figure 6A and 6C whereas the survival curves are represented in Figure 6B and 6D. In mice vaccinated with empty control vector, footpad size started to increase 5 weeks after *M. ulcerans* infection whereas in BCG vaccinated mice, footpad swelling was delayed for 7–8 weeks (Figures 6A and 6C). Vaccination with DNA encoding Ag85A from *M. tuberculosis* or from *M. ulcerans* delayed onset of foot pad swelling by only 2 to 3 weeks (Figure 6A). DNA prime/protein boost protocol using the *M. tuberculosis* Ag85A did not increase vaccine efficacy (Figure 6C) whereas vaccination with DNA encoding Ag85A from *M. ulcerans* boosted with the recombinant Ag85A-MPL-A protein from *M. ulcerans* delayed onset of foot pad swelling to the same extent as the BCG vaccine by 7 to 8 weeks (Figure 6C). Survival curves reflected the footpad swelling pattern. Median survival time of mice vaccinated with empty vector was 10.5 weeks, whereas BCG vaccination delayed significantly the moment when mice had to be euthanized, resulting in a median survival time of 17.5 weeks (Figures 6B and 6D) (p<0.001 compared to empty vector vaccinated mice; p<0.01 compared to 85A-DNA Mu vaccinated mice). *M. ulcerans* DNA vaccinated mice demonstrated a median survival time of 13 weeks (p<0.01 compared to empty vector vaccinated mice according to the log rank test). Similar results were observed in mice vaccinated with DNA encoding Ag85A from *M. tuberculosis* (median survival time 12.5 weeks) (Figure 6B). Boosting DNA vaccinated mice with protein from *M. tuberculosis* did not increase protective efficacy of the DNA vaccine but priming with DNA encoding Ag85A from *M. ulcerans* and boosting with recombinant Ag85A from *M. ulcerans* was very effective in increasing the protection (Figure 6D) (p<0.001 compared to *M. ulcerans* DNA alone, p<0.01 compared to DNA encoding Ag85A from *M. tuberculosis* boosted with the protein of *M. tuberculosis*). Median survival time in the *M. ulcerans* DNA primed- *M. ulcerans* protein boosted mice was 17 weeks. This protection was comparable to that conferred by BCG (p>0.05).

## Discussion

Buruli ulcer belongs to the family of neglected tropical diseases [48]. In 1998 the first International Conference on Buruli Ulcer was organized in Côte d'Ivoire, expressing the poor knowledge about this disease and calling on the international scientific community to support control and research efforts. Currently, no specific vaccine exists against this disease. In 1957, Fenner demonstrated that a high degree of protection was conferred, in an experimental mouse model, against challenge infection with small doses of *M. ulcerans* by prior inoculation with *M. ulcerans*, *M. balnei* and *M. bovis* BCG (BCG). Footpad and intravenous BCG administration gave considerable protection against a small dose and a slight protection against a large dose of *M. ulcerans* given in the other footpad [49]. More recently we have shown in a similar experimental mouse model that BCG vaccine protects to some extent against infection with *M. ulcerans* but that a booster vaccination with the same BCG vaccine does not increase the protective effect [31],[47]. In 1969, a clinical study performed in Uganda reported on a protection rate of 47% of the BCG vaccine. However, protection turned out to be short-lived and was only detected in the first 6 months following BCG vaccination [24]. In 1976, Smith *et al* reported another BCG vaccination trial against Buruli ulcer in Uganda giving similar short lived (one year) protection rates of about 50% [25]. Although not very effective at preventing the classical skin lesions of Buruli ulcer, the BCG vaccine seems to exert a significant protective effect against its severe, disseminated osteomyelitis form both in children and in adults [26],[27].

A more effective *M. ulcerans* vaccine would certainly help to control this debilitating disease that affects particularly children. Unfortunately, the nature of the protective immune response and the precise antigens involved are not fully defined at the moment. Based on biopsy specimens, *M. ulcerans* was originally thought to reside exclusively as free extracellular bacilli, implying that humoral responses might be protective. However, Coutanceau *et al* recently demonstrated that the initial phase of *M. ulcerans* infection proceeds by internalization of bacilli by phagocytic cells and that the extracellular stage results from mycolactone inducing host cell death [6],[50]. Therefore, recognition of the early intracellular stage by an effective Th1 type immune response may contribute to the control of the infection, that is in so far as it can help to reduce the mycolactone production. Hence, magnitude of mycobacteria-specific Th1 type immune response is a plausible correlate of protection that can be used to analyze the potential of new, experimental vaccines.

In this study, we focused on a plasmid DNA vaccine encoding Ag85A from *M. ulcerans.* Protective efficacy was evaluated using two approaches, in one experiment by enumerating the number of AFB in the footpad at nine weeks after *M. ulcerans* challenge and in the other experiment by monitoring footpad swelling and long term survival of the mice. We have previously reported that footpad swelling is correlated with bacterial replication and can be used as an alternative read-out for protection against infection [47]. DNA prime–protein boost strategy using specific *M. ulcerans* antigen 85A was clearly the most effective, reducing about one hundred fold the bacterial number and offering a protection of comparable magnitude as the one induced by the BCG vaccine. Nevertheless, and as for the BCG vaccine, immune protection was not sterilizing and eventually all mice developed footpad swelling. We hypothesize that the vaccines reduced or delayed temporarily mycolactone production by the virulent type 1 strain 04-855 but that immunity was not strong enough to completely block the ML synthesis. Targeting ML production by specific antibodies or by interfering with its synthesis might help to overcome this problem. A study made by Fenner, in 1956 showed that the apparition of footpad swelling depends of the number of viable AFB in the inoculum, small doses of bacilli showing delayed appearance of footpad lesion [51]. As we used a high inoculum size of 10^5^ AFB in our studies, it is possible that more sustained protections could have been observed if we had administered a lower number of bacteria.

The Gerbu adjuvant is less well known as immunomodulator than other adjuvants such as alumn or monosphoshoryl-lipd-A (MPL-A) [52]. Here we have shown that this adjuvant has a strong Th1 inducing capacity, as indicated by the elevated levels of antigen-specific IL-2 and IFN-γ that could be detected in spleen cell cultures from mice vaccinated with protein in this adjuvant. Antibodies of both IgG1 but also of IgG2b isotype were induced, which was another indication of its Th1 favouring properties. Vaccination with recombinant *M. ulcerans* Ag85A protein in Gerbu adjuvant induced comparable Th1 cytokine and antibody levels as the prime-boost DNA vaccination. This protein vaccine also induced considerable protection (albeit somewhat lower that the DNA based vaccine) as indicated by significantly reduced number of AFB in the footpad at nine weeks after *M. ulcerans* challenge. We have previously shown that DNA vaccination induces a broader T cell repertoire (more protein epitopes recognized) than infection with tuberculosis [53],[54], vaccination with BCG [44] or with protein [46] and this may explain the better protection conferred by the DNA prime-protein boost vaccination. It is also possible that immune memory induced with this combined immunization protocol was stronger and longer lasting than immune memory induced with protein only vaccination.

Analysis of the H-2^b^ restricted Th1 T cell epitopes of antigen 85A from *M. ulcerans* and from *M. tuberculosis* revealed some extent of species specificity, both in the NH_2_-terminal and in the COOH-terminal half of the protein. In contrast to the response induced with DNA encoding *M. tuberculosis* Ag85A, which was preferentially directed against Ag85A peptide spanning aa 261–280, T cell response induced with DNA encoding the *M. ulcerans* protein was directed preferentially against peptide spanning aa 240–259. Remarkably, mice vaccinated with the *M. tuberculosis* DNA reacted more strongly to this peptide region of *M. ulcerans* (25,000 cpm of IL-2/5,000 pg of IFN-γ) than to the same region in *M. tuberculosis* (10,000 cpm of IL-2/1,000 pg of IFN-γ). We have previously reported that B6 mice vaccinated with DNA encoding Ag85B from *M. tuberculosis* also react more strongly to 85B peptide spanning aa 244–260 than to peptide spanning aa 262–279 [46]. Sequence analysis of the 241–260 region of Ag85 revealed that the Ag85A sequence from *M. ulcerans* is more similar to the Ag85B sequence of *M. tuberculosis* (only 1 aa (A–D) change in position 242) than to the Ag85A sequence of *M. tuberculosis* (4 aa changes). Interestingly, it was demonstrated by Yanagisawa *et al* that vaccination of B6 mice with killed *M. tuberculosis* triggered preferentially a v_β_11+ CD4+ T cell response against the peptide spanning amino acids 240 to 254 of Ag85B [55]. All these data taken together seem to indicate that the *M. ulcerans* Ag85A_241–260_ region is more immunogenic than the corresponding *M. tuberculosis* Ag85A region and this may explain the better protective efficacy that we have observed with the species specific *M. ulcerans* vaccine.

In conclusion, our results show that specific Ag85A-DNA priming followed by protein boosting is an effective way to induce robust Th1 type immune responses and strong protection against experimental footpad infection with *M. ulcerans* in mice. This is a promising vaccination approach that warrants further analysis. Combination with vaccines targeting mycolactone or with vaccines targeting enzymes involved in mycolactone synthesis may be a way to strengthen its protective efficacy.
